# Emerging Roles of Bile Acids in Neuroinflammation

**DOI:** 10.3390/ijms262311301

**Published:** 2025-11-22

**Authors:** Erika L. Butcher, Subha Arthur

**Affiliations:** Department of Biomedical Sciences, Marshall University Joan C. Edwards School of Medicine, Huntington, WV 25755-0001, USA; butcher111@marshall.edu

**Keywords:** bile acids, neuroinflammation, blood-brain barrier, FXR, TGR5, neurodegeneration, Parkinson’s disease, Alzheimer’s disease, multiple sclerosis, Huntington’s disease, amyotrophic lateral sclerosis

## Abstract

Bile acids, once considered mere digestive detergents, have emerged as multifaceted signaling molecules with systemic influence extending far beyond the gastrointestinal tract. Recent discoveries reveal their capacity to modulate immune responses, cross the blood–brain barrier, and interact with central nervous system (CNS) cells through their receptors. Neuroinflammation, a key driver of neurodegenerative and neuroimmune disorders, is increasingly linked to bile acid signaling pathways that regulate glial activation, cytokine production, and neuronal survival. This review compiles the current evidence connecting bile acids to CNS inflammation, highlighting mechanistic insights, disease-specific alterations, and the gut–microbiome-bile acid-brain axis. It also explores the therapeutic potential of bile acid derivatives and receptor modulators, as well as their emerging role as biomarkers in conditions such as Alzheimer’s disease, multiple sclerosis, and hepatic encephalopathy. Despite promising advances, critical gaps remain, including the need for bile receptor mapping in human CNS cells, standardized CNS bile acid profiling, and longitudinal metabolomic studies. Bridging these gaps may unlock new strategies for targeting neuroinflammation through bile acid-immune crosstalk.

## 1. Introduction

Formerly considered only as digestive surfactants aiding in the absorption of dietary fats, bile acids have emerged over the last decade as dynamic regulators of metabolism. These amphipathic molecules, produced as end products of cholesterol catabolism in the liver, are now recognized for their potent signaling capabilities influencing an array of cellular, physiological and immunological processes [1,2,3]. This significant evolution of the understanding of their systemic functions evolved dramatically after the discovery of bile acid activated receptors. Bile acids modulate diverse functions primarily by interacting with nuclear receptors such as the Farnesoid X Receptor (FXR) and membrane-bound receptors like Takeda G-protein-coupled receptor 5 (TGR5) [4,5,6,7,8,9,10,11,12,13,14]. Activation of FXR in the liver and intestine regulates transcription of genes involved in bile acid synthesis, lipid metabolism, and glucose homeostasis, thereby contributing to metabolic homeostasis. In parallel, TGR5 expressed in tissues including brown adipose tissue, intestinal epithelium, and immune cells, initiates signaling cascades that enhance energy expenditure, improve insulin sensitivity, and modulate inflammatory responses. Through these receptor-mediated pathways, bile acids orchestrate a complex network of metabolic and immunological processes, positioning them as key regulators of nutrient signaling and systemic homeostasis.

Compelling evidence now suggests that bile acids possess the ability to cross the blood–brain barrier, a highly selective membrane that typically restricts the entry of peripheral substances into the central nervous system (CNS) [15,16,17]. Once within the CNS, bile acids may interact with neural receptors and signaling pathways, influencing processes such as neuroinflammation, neurotransmitter regulation, and neuronal energy metabolism [18,19,20]. Studies have identified specific bile acid transporters and receptors in brain tissue, indicating a direct role in modulating brain function [21]. This emerging neurobiological role suggests bile acids as potential mediators in neurological disorders, including Alzheimer’s disease, Parkinson’s disease, and hepatic encephalopathy [22,23,24,25,26,27,28,29]. As research continues to uncover the mechanisms behind these interactions, bile acids are increasingly viewed not just as digestive agents, but as integral components of the gut–brain axis with far-reaching implications for neurophysiological processes and the regulation of neuroinflammation.

Neuroinflammation is being progressively acknowledged as a pivotal mechanism in the pathogenesis of numerous neurological disorders. Whether in neurodegenerative diseases like Alzheimer’s and Parkinson’s, or neuroimmune conditions such as multiple sclerosis, chronic activation of glial cells and sustained release of pro-inflammatory cytokines contribute to neuronal dysfunction and degeneration. Microglia and astrocytes, the primary immune cells of the CNS, play dual roles, protective in acute injury but deleterious when chronically activated. Understanding the upstream modulators of these inflammatory responses is critical for developing targeted therapies.

With the growing body of research on bile acid signaling and its systemic effects, this review will focus on connecting bile acid biology to neuroinflammatory processes. The mechanistic interplay between bile acid signaling and neuroinflammation has emerged as a compelling area of research, revealing how these metabolic molecules transcend their classical roles in digestion to act as neuromodulators. By influencing glial activation, cytokine dynamics, and neuronal survival, bile acids seemingly play a pivotal role in shaping neuroinflammatory outcomes. This review will address current evidence linking bile acid biology to CNS immune responses, with particular emphasis on their therapeutic potential in neurodegenerative and neuroinflammatory disorders.

## 2. Bile Acids: Multifunctional Molecules in Digestion and Metabolic Regulation

Bile acids are a major component of bile with a crucial role in the digestion and absorption of dietary fats and fat-soluble vitamins in the small intestine. Synthesized in hepatocytes as terminal products of cholesterol catabolism, bile acids are secreted into the bile canaliculi, stored in the gallbladder, and released into the small intestine after a meal. Their amphipathic nature enables them to emulsify lipids and form micelles that facilitate the action of pancreatic lipases for efficient fat digestion and absorption in the small intestine. The majority of bile acids secreted in the proximal small intestine to aid in the digestion of fats are reabsorbed in the ileum and returned to the liver via the enterohepatic circulation, a highly efficient recycling system that conserves resources and regulates bile acid synthesis. It not only conserves metabolic resources but also tightly regulates bile acid synthesis through feedback mechanisms involving nuclear receptors like FXR. By sensing bile acid concentrations, FXR modulates gene expression to suppress further synthesis, maintaining homeostasis and preventing hepatotoxicity [2,30,31,32].

Beyond their classical digestive functions, bile acids are now recognized as potent metabolic regulators. They act as signaling molecules by engaging nuclear receptors such as FXR and membrane-bound receptors like TGR5. These interactions influence a wide array of physiological processes, including glucose metabolism, lipid regulation, energy expenditure, and modulation of inflammatory pathways. For instance, FXR activation improves insulin sensitivity and reduces hepatic triglyceride accumulation, while TGR5 stimulation enhances energy expenditure and exerts anti-inflammatory effects [33,34]. Additionally, bile acids possess antimicrobial properties, disrupting bacterial membranes and contributing to gut microbial homeostasis, an essential aspect of immune defense and nutrient absorption [35,36].

Bile acids are categorized into primary, secondary, and tertiary types. Primary bile acids cholic acid (CA) and chenodeoxycholic acid (CDCA) are synthesized in the liver and typically conjugated with glycine or taurine to increase solubility. Primary bile acids that reach the colon are modified by gut microbiota, impacting their chemical diversity and biological activity. Gut microbiotas modify primary bile acids into secondary bile acids such as deoxycholic acid (DCA) and lithocholic acid (LCA), which are more hydrophobic and potentially cytotoxic. Tertiary bile acids, like ursodeoxycholic acid (UDCA) and their modified forms, are known to have anticholestatic, antioxidant, anti-inflammatory, antiapoptotic, and immunomodulatory properties and are therefore used particularly for treating cholestatic liver diseases and metabolic disorders [30,37,38,39].

The dynamic interplay between bile acid metabolism, gut microbiota, and host physiology underscores a complex regulatory network that extends far beyond digestion. Disruptions in bile acid homeostasis, due to liver dysfunction, intestinal disease, or microbial imbalance, can contribute to a spectrum of health issues, including non-alcoholic fatty liver disease, insulin resistance, inflammatory bowel disease, and even colorectal cancer [40]. Therefore, understanding these interconnected systems is crucial for developing targeted therapies and promoting overall health.

## 3. Bile Acids as Neuroimmune Signaling Molecules

**Figure 1:**
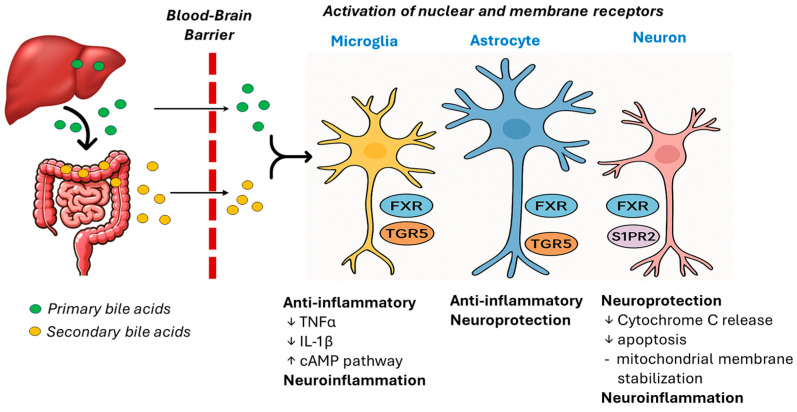
Bile acid signaling in the cells of the CNS. Primary bile acids synthesized in the liver and secondary bile acids modified by bacteria in the gut can cross the blood–brain barrier and interact with nuclear (FXR) and membrane (TGR5, S1PR2) receptors expressed on microglia, astrocytes, and neurons. Bile acids and their downstream signaling processes either promote neuroinflammatory processes or regulate anti-inflammatory and neuroprotective processes in the CNS cells (↑ indicates increases and ↓ indicates decreases).

Priorly considered confined to enterohepatic circulation, bile acids are now recognized as versatile endocrine-like molecules capable of influencing distant organs, including the CNS. Their signaling capacity is mediated through a diverse array of receptors, both canonical and non-canonical, that orchestrate metabolic and immunological responses across tissues, including within the brain. Among the canonical receptors, FXR and G protein-coupled bile acid receptor 1 (TGR5; also called GPBAR1 (G-protein coupled bile acid receptor 1)) are the most extensively studied for their roles in neuroinflammation. FXR, a nuclear receptor primarily expressed in the liver, intestine, and kidneys, also exhibits functional relevance in the CNS. It regulates bile acid synthesis and metabolic homeostasis while exerting anti-inflammatory effects by suppressing NF-κB signaling and modulating cytokine production [41,42,43,44]. These actions suggest FXR could help mitigate neuroinflammatory cascades associated with neurodegenerative diseases. TGR5, a membrane-bound receptor, is expressed in a broader range of tissues, including immune cells and specific cells of the brain, such as microglia and astrocytes. Activation of TGR5 elevates intracellular cyclic AMP (cAMP) levels, promoting anti-inflammatory signaling and energy expenditure [45]. In microglia, TGR5 stimulation has been shown to attenuate the release of pro-inflammatory cytokines, making it a potential therapeutic target for controlling neuroinflammation and preserving neuronal integrity [46,47,48,49,50,51,52]. Beyond these canonical receptors, bile acids also engage non-canonical targets such as sphingosine-1-phosphate receptor 2 (S1PR2). This receptor mediates bile acid-induced signaling in endothelial and neural cells, influencing vascular tone, cell migration, and neuroimmune interactions [53]. S1PR2 activation may contribute to the regulation of blood–brain barrier permeability and immune cell trafficking, further implicating bile acid signaling in CNS inflammatory dynamics. Together, these receptors underscore the complexity of bile acid signaling and its emerging relevance in modulating neuroinflammatory processes, offering promising avenues for therapeutic intervention in inflammatory CNS disorders (Figure 1).

## 4. Bile Acids and the Blood-Brain-Barrier

Bile acids are being increasingly implicated as powerful modulators of brain function, with their ability to cross the blood–brain barrier (BBB) opening new avenues in neuroimmune and neurodegenerative research. Recent studies have illuminated a paradigm shift in our understanding of bile acids as bioactive molecules with far-reaching effects on the CNS. Evidence now confirms that bile acids can traverse the BBB, appearing in cerebrospinal fluid (CSF) and brain tissue under both normal and disease states [20,49,54,55]. This translocation is facilitated by organic anion transport polypeptides (OATP) and passive diffusion, depending on the physicochemical properties of the bile acid species involved. Once inside the CNS, bile acids engage with a variety of receptors, including FXR and TGR5, which are expressed in neurons, astrocytes, and microglia [49]. These interactions suggest that bile acids may function as neuromodulators, influencing neurotransmitter release, synaptic plasticity, and glial cell activity. For instance, tauroursodeoxycholic acid (TUDCA) has demonstrated neuroprotective effects by reducing oxidative stress, inhibiting apoptosis, and modulating inflammatory cytokine production in neuroinflammatory disorders [20,56].

Implications of bile acid-mediated neuroimmune regulation seem to be profound. Bile acids may help orchestrate the balance between pro- and anti-inflammatory signaling in the brain, potentially impacting conditions such as multiple sclerosis, Alzheimer’s disease, and Parkinson’s disease. Dysregulated bile acid metabolism, whether due to liver dysfunction, altered gut microbiota, or genetic factors, could disrupt CNS homeostasis, contributing to neuroinflammation and neurodegeneration [57]. Moreover, the discovery of endogenous bile acid synthesis within the brain, via enzymes like CYP46A1, adds another layer of complexity [58,59]. This suggests that the CNS may not only receive bile acids from peripheral circulation but also produce them locally for specific signaling functions [20]. Therefore, bile acids are no longer confined to the realm of hepatic and gastrointestinal physiology, rather recognized as integral components of the gut–liver–brain axis, with the potential to reshape our understanding of brain health and disease. As research continues to unravel their molecular pathways and therapeutic potential, bile acids will most likely become central figures in the next generation of neurobiological discovery.

## 5. Mechanistic Links Between Bile Acids and Neuroinflammation

Bile acids exert both pathological and beneficial effects in neuroinflammation, highlighting the importance of maintaining bile acid homeostasis to prevent or mitigate neuroimmune disorders. The mechanistic interplay between bile acid signaling and neuroinflammation is increasingly supported by mechanistic evidence at both cellular and molecular levels. Bile acids, particularly their conjugated and secondary forms, engage with cells in the CNS and modulate inflammatory pathways central to neurodegenerative and neuroimmune disorders.

The neuroimmune landscape of the CNS is shaped by dynamic interplay between resident cells and signaling molecules, among which bile acids have emerged as potent modulators. Hydrophilic bile acid TUDCA, a taurine conjugate of UDCA, exerts anti-inflammatory and cytoprotective effects across multiple CNS cell types, influencing both innate immune responses and neuronal survival. UCDA’s glycine conjugate glycoursodeoxycholic acid (GUDCA) alters mitochondria dynamics and apoptosis by reducing caspase-9 levels in motor neuron-like cells used in amyotrophic lateral sclerosis studies [60]. Microglia, the brain’s primary immune sentinels, play a central role in initiating and sustaining neuroinflammation. TUDCA has been shown to attenuate microglial activation by suppressing the expression of pro-inflammatory cytokines such as TNF-α and IL-1β, and by inhibiting polarization toward the M1 phenotype, associated with neurotoxic responses. Moreover, TUCDA supplementation reduced neuroinflammation and altered microglial behavior in models of multiple sclerosis [61]. Additionally, TUDCA acts as an agonist of the bile acid receptor TGR5, which is expressed on microglia and mediates anti-inflammatory signaling through cAMP-dependent pathways [44]. Neurons, although not traditionally considered immune cells, are highly susceptible to inflammatory insults. TUDCA has demonstrated robust neuroprotective effects by stabilizing mitochondrial membranes, reducing cytochrome c release, and inhibiting caspase-mediated apoptosis [24]. These actions preserve neuronal integrity under conditions of oxidative stress and excitotoxicity. TUDCA in combination with coenzyme Q10 and creatine provided an additive neuroprotective effect in in vitro models of Parkinson’s disease, highlighting its potential in neurodegenerative contexts [62]. Collectively, these findings underscore the multifaceted role of bile acids, especially TUDCA, in modulating CNS inflammation and neurodegeneration. By targeting microglia, astrocytes, and neurons through receptor-mediated and mitochondrial pathways, bile acids like TUDCA offer promising therapeutic strategies for conditions such as multiple sclerosis, Alzheimer’s disease, and Parkinson’s disease.

At the molecular level, bile acids exert anti-inflammatory effects primarily through activation of the nuclear receptor FXR and G-protein-coupled receptor TGR5, both of which suppress the NF-κB signaling pathway, a central regulator of pro-inflammatory gene expression [63,64]. This inhibition leads to decreased transcription of cytokines, chemokines, and adhesion molecules that drive neuroinflammatory cascades. Bile acids also reduce endoplasmic reticulum (ER) stress and the accumulation of reactive oxygen species (ROS), both of which are implicated in neuronal and glial dysfunction. This crosstalk between bile acid signaling and cellular stress pathways supports cellular homeostasis and protects against inflammation-induced cellular damage, reinforcing the therapeutic potential of bile acids in neuroimmune disorders.

Although the beneficial effects of bile acids are well recognized in the context of neuroinflammation, some species of bile acids have been noted for their pathophysiological processes of neuroinflammation (Table 1). Dysregulation of bile acid synthesis or composition, often stemming from liver dysfunction or gut microbiota imbalance, can lead to the accumulation of neurotoxic bile acid species that cross the blood–brain barrier and activate microglia and astrocytes, triggering chronic inflammation [21]. This inflammatory cascade is implicated in the pathogenesis of disorders like Alzheimer’s disease, Parkinson’s disease, and multiple sclerosis [20]. Moreover, altered bile acid profiles can impair mitochondrial function and increase oxidative stress, further exacerbating neuronal damage [57]. The brain–liver–gut axis plays a pivotal role in this process, with bile acid signaling acting as a key mediator of communication between peripheral organs and the CNS [65]. The pathogenic mechanisms caused by specific bile acids in neurological and neuroimmune disorders are given in Table 1.

## 6. Gut–Microbiome–Bile Acid–Brain Axis and Neuroinflammation

The gut–microbiome–bile acid–brain axis represents a dynamic and multifaceted communication network with a critical role in regulating neuroinflammation. Gut microbiota metabolize primary bile acids into secondary bile acids, which act as signaling molecules through receptors such as FXR and TGR5 [18]. These bile acid–receptor interactions influence systemic immune responses, oxidative stress, and blood–brain barrier integrity. Alterations in gut microbial composition or bile acid profiles, often seen in dysbiosis, can disrupt this signaling, leading to heightened microglial activation and increased production of pro-inflammatory cytokines in the CNS. Such disruptions are implicated in the pathogenesis of neurodegenerative diseases, including Alzheimer’s, Parkinson’s, and multiple sclerosis [22,23,24,25,26,27,28,29,74]. Of particular interest is the generation of neuroactive bile acids through microbial metabolism. Secondary bile acids such as DCA and LCA can cross the blood–brain barrier and interact with CNS receptors, influencing neuroinflammatory pathways, glial cell activation, and neuronal signaling [20,21,49,57,75]. Moreover, gut microbiota-mediated bile acid dysregulation, particularly reduced taurodeoxycholic acid (TDCA), contributes to Androgen Deprivation Therapy (ADT)-induced cognitive dysfunction via impaired TGR5-ERK1/2 signaling [76]. These interactions suggest a mechanistic link between gut microbial activity and CNS homeostasis, with implications for neurodegenerative and neuropsychiatric disorders. Furthermore, age-related changes in the gut microbiome and hepatic function contribute to altered bile acid profiles. Notably, the accumulation of tauro-β-muricholic acid (TβMCA), a bile acid with antagonistic effects on FXR, has been implicated in promoting microglial activation and neuroinflammation [77]. This shift in bile acid composition during aging may exacerbate CNS immune dysregulation, thereby contributing to cognitive decline and the pathogenesis of neurodegenerative diseases.

Therapeutically, modulating this axis offers promising avenues for intervention. Strategies such as microbiota-directed therapies (e.g., probiotics, prebiotics), bile acid receptor agonists, and dietary modifications will aim to restore microbial balance and bile acid homeostasis, subsequently mitigating neuroinflammatory responses. Continued research into the mechanistic links between gut-derived metabolites and brain immune signaling may yield novel biomarkers and targeted treatments for neuroinflammatory and neurodegenerative disorders.

## 7. Bile Acids in Neurological and Neuroimmune Disorders

The role of bile acids in CNS pathology is increasingly recognized across a spectrum of neurological diseases. Alterations in bile acid metabolism, signaling, and transport have been implicated in neurodegeneration, neuroinflammation, and cognitive decline. This section explores how bile acid dysregulation contributes to disease progression and how specific bile acid derivatives may offer therapeutic potential.

### 7.1. Alzheimer’s and Parkinson’s Disease

Neurodegenerative diseases such as Alzheimer’s and Parkinson’s are characterized by progressive neuronal loss, protein aggregation, and chronic neuroinflammation. Bile acids are increasingly recognized as key modulators in both Alzheimer’s and Parkinson’s disease, influencing neurodegeneration through gut–brain signaling, metabolic dysfunction and inflammation. Recent literature highlights a compelling link between bile acid metabolism and the pathogenesis of both Alzheimer’s disease and Parkinson’s disease. In Alzheimer’s disease, altered bile acid profiles, particularly elevated secondary bile acids like DCA, TDCA and glycodeoxycholic Acid (GDCA), have been associated with increased amyloid-beta deposition and tau pathology [78]. These bile acids can cross the blood–brain barrier and activate receptors such as FXR and TGR5, modulating neuroinflammation and neuronal survival. Similarly, in Parkinson’s disease patients, lower levels of CA, CDCA and UDCA were significantly associated with mild cognitive impairment, suggesting that bile acid dysregulation may contribute to non-motor symptoms and cognitive decline [79]. Moreover, bile acid analysis in the appendix of patients with Parkinson’s disease reveals an increase in hydrophobic and secondary bile acids such as DCA and LCA, suggesting that biliary abnormalities may play a role in the pathogenesis of Parkinson’s disease and the potential of these bile acids to serve as early biomarkers. Moreover, bile acid TUDCA has shown neuroprotective effects in preclinical models of both diseases by not only reducing inflammation but also reducing oxidative stress and preventing apoptosis, enhancing glucose homeostasis, making it a potential therapeutic for neurodegenerative diseases like Alzheimer’s and Parkinson’s, diseases [24,80,81]. Additionally, the bile acid UDCA which is known to cross the blood–brain barrier, has shown promise as a neuroprotective agent. Its ability to stabilize mitochondrial membranes, reduce oxidative stress, inhibit apoptosis and more importantly, reduce inflammation, positions it as a candidate for therapeutic intervention for Alzheimer’s and Parkinson’s disease [82,83,84]. In preclinical models, UDCA has improved neuronal survival and reduced neuroinflammatory markers, offering therapeutic scope in slowing disease progression [85,86]. Additionally, recent metabolomic and transcriptomic studies have revealed significant alterations in circulating, CSF and brain bile acid profiles in affected individuals, including increased levels of secondary bile acids and decreased neuroprotective species, suggesting a disruption in bile acid homeostasis and gut dysbiosis [66,86,87,88].

The accumulating evidence underscores the multifaceted role of bile acids in the pathophysiology of Alzheimer’s and Parkinson’s disease, positioning them as both biomarkers and therapeutic targets in neurodegeneration. Their ability to modulate neuroinflammation, oxidative stress, and mitochondrial integrity through gut–brain axis signaling and receptor-mediated pathways highlights their systemic influence beyond hepatic metabolism. The consistent findings of elevated neurotoxic secondary bile acids and reduced neuroprotective species in affected individuals, coupled with promising preclinical outcomes from bile acid-based interventions such as TUDCA and UDCA, suggest that restoring bile acid homeostasis could offer a novel strategy for mitigating disease progression. As metabolomic and transcriptomic technologies advance, further elucidation of bile acid dynamics may pave the way for precision medicine approaches in the diagnosis, monitoring, and treatment of neurodegenerative disorders.

### 7.2. Multiple Sclerosis

Multiple sclerosis (MS) is a chronic autoimmune disorder marked by demyelination and neuroinflammation. Recent literature underscores the pivotal role of bile acid metabolism and receptor signaling in the pathophysiology of this disease. Studies reveal MS patients exhibit significantly altered bile acid profiles in both serum and CSF, correlating with disease severity and immune dysfunction [89,90]. These disruptions reinforce that bile acids are not merely digestive agents but active participants in neuroimmune regulation. A few studies also demonstrated that bile acid metabolism is significantly altered in both serum and CSF of MS patients and the changes correlate with disease severity and immune dysregulation [61,89].

In experimental autoimmune encephalomyelitis (EAE), a mouse model of MS, TUDCA treatment mitigated astrocytic neuroinflammation by regulating TGR5-mediated AKT/NFκB signaling pathway, thus alleviating EAE [39]. In another EAE study, oral treatment with obeticholic acid (6α-ethyl-chenodeoxycholic acid, 6-ECDCA), a synthetic FXR agonist, identified FXR as a negative regulator of neuroinflammation, suggesting FXR agonists might serve as a potential therapy compound for MS [43]. In a mouse model of MS, UDCA supplementation downregulated MS progression by modulating microglial activity via TGR5 in the spinal cord [91]. In this study, proteomic analysis of UDCA-treated activated microglial (MG6) cells revealed that the TGR5 inhibitor significantly decreased the expression of 6 anti-neuroinflammatory proteins: A2M, AHSG, ALB, APOA1, APOH, and SPP2. The potential for targeting TGR5 in alleviating MS was also demonstrated in another study, where treatment with GPBAR1/TGR5 small molecule agonist exhibited a significant reduction in the EAE clinical score, which correlated with reduced monocyte and microglial activation, as well as reduced trafficking of monocytes and T cells into the CNS [92]. Most importantly, bile acid supplementation appears to be safe and well-tolerated in progressive MS, with early trials suggesting beneficial outcomes [61,70].

This emerging evidence highlights bile acid metabolism and receptor signaling, particularly through FXR and TGR5, as critical modulators of neuroinflammation and immune dysregulation in multiple sclerosis. Therapeutic interventions using bile acid derivatives such as TUDCA, UDCA, and synthetic FXR agonists have demonstrated promising results in preclinical models, showing reduced inflammatory responses, enhanced remyelination, and improved clinical outcomes. The safety and tolerability of bile acid supplementation in progressive MS further support its potential as an adjunctive therapeutic strategy. These findings pave the way for future clinical exploration of bile acid-based therapies in MS management.

### 7.3. Hepatic Encephalopathy

Hepatic encephalopathy (HE) arises from liver dysfunction, characterized by cognitive impairment, motor disturbances, and altered consciousness due to the accumulation of toxic bile acids in systemic circulation and the CNS. A significant feature of HE is microglial activation and an increase in neuroinflammation, where bile acids seem to play a significant role. Studies in a mouse model of HE have shown that activation of bile acid non-canonical receptor S1PR2 signaling by conjugated bile acids such as taurocholic acid (TCA) promotes neuroinflammation [53]. A rat study demonstrated that bile acids exacerbated the progression of HE [51]. However, oral cholestyramine (a bile acid chelator) treatment mitigated the progression of HE. This was accompanied by reduced total bile acid content in the serum and cerebral cortex, decreased levels of pro-inflammatory cytokines such as IL-1β and IL-6, increased levels of the anti-inflammatory factor IL-10, and increased expression of the TGR5 receptor in the cerebral cortex.

These findings underscore the importance of bile acid detoxification and transport in maintaining CNS homeoyoxystasis. Therapeutic strategies aimed at reducing toxic bile acid levels or enhancing protective bile acid signaling by regulating S1PR2 or TGR5 may mitigate neurological symptoms in HE.

### 7.4. Aging and Cognitive Decline

Aging is increasingly recognized as a key modulator of gut microbiota composition and bile acid metabolism, with profound implications for brain health [93]. Studies have shown that the aging gut undergoes microbial dysbiosis, leading to altered bile acid profiles, including increased levels of neuroactive bile acids such as TβMCA [77]. These bile acids can cross the blood–brain barrier and interact with cells in the CNS, influencing neuroimmune signaling pathways. TβMCA acts as an antagonist of FXR, a nuclear receptor involved in regulating inflammation and bile acid homeostasis. Its accumulation in aged individuals has been linked to microglial activation, a hallmark of neuroinflammation, which contributes to synaptic dysfunction and cognitive decline. This pro-inflammatory environment exacerbates neuronal damage and impairs memory, as observed in both animal models and human studies of neurodegenerative diseases. Consequently, the gut-brain-bile acid axis has emerged as a promising therapeutic target, with interventions aimed at modulating microbiota composition, bile acid signaling, and neuroimmune responses offering potential to preserve cognitive function in aging populations.

### 7.5. Amyotrophic Lateral Sclerosis

Recent studies increasingly implicate bile acids in the pathophysiology and potential treatment of Amyotrophic Lateral Sclerosis (ALS), a disease of progressive deterioration and loss of function of motor neurons in the brain and spinal cord. Bile acids such as UDCA and TUDCA have demonstrated beneficial effects in ALS [54]. In clinical studies, oral treatment of UDCA has been shown to enter the CSF and cross the BBB in a dose-dependent manner and slow the rate of progression in ALS patients. Moreover, patients showed excellent safety and tolerability of UDCA, but with adverse gastrointestinal effects [54,55,94]. Similarly, in a study in the hSOD1^G93A^ mouse model of ALS, delayed muscle denervation and a slower progression in ALS were observed after TUDCA administration [95]. A 2024 case–control study found elevated serum levels of UDCA, TUDCA, and GUDCA in ALS patients, particularly in familial cases, suggesting altered bile acid metabolism rather than deficiency [96]. GUDCA has also been shown to inhibit apoptosis in an in vitro model of ALS, the motor neuron-like NSC-34 cells carrying the hSOD1^G93A^ mutation [60]. In this study, GUDCA treatment prevented changes in mitochondrial dynamic properties and apoptosis by reducing caspase-9 levels, indicating its beneficial anti-apoptotic effects required to decelerate the onset and progression of ALS. Clinically, TUDCA has been evaluated in the TUDCA-ALS Phase III trial, which aimed to assess its efficacy as an add-on to riluzole; although the trial did not meet its primary endpoint, real-world data suggest that higher doses of TUDCA may extend survival in ALS patients [97]. Additionally, rare cases of ALS co-occurring with primary biliary cirrhosis (PBC) hint at shared mitochondrial and immune dysfunctions between the two diseases [98]. UDCA, as an immunomodulatory agent, has a protective role on cholangiocytes against bile acid toxicity in PBC patients and also has a therapeutic effect on ALS, thus indicating UDCA as a common beneficial factor in both ALS and PBC. These findings highlight the therapeutic potential of bile acids in ALS and underscore the importance of further investigation of bile acid signaling and metabolism as both biomarkers and treatment targets.

### 7.6. Huntington’s Disease

Recent studies unveiled the involvement of bile acids in pathophysiology and potential treatment of Huntington’s disease (HD), a progressive neurodegenerative disorder caused by CAG repeat expansions in the *HTT* gene [99]. As early as 2001, a study in a rat model of HD showed that the delivery of TUDCA to the brain and demonstrated its neuroprotective effect [100]. TUDCA administration protected rats against motor and cognitive deficits and reduced striatal degeneration in the 3-nitropropionic acid model of HD. In an R6/2 transgenic mouse model of HD, TUDCA treatment reduced striatal atrophy, decreased striatal apoptosis and reduced the size of ubiquitinated neuronal intranuclear huntingtin inclusion aggregates. Moreover, locomotor and sensorimotor deficits were significantly improved in the TUDCA-treated HD mice model [101].

Metabolomic profiling of HD patients revealed significant alterations in plasma bile acid levels. HD patients exhibited higher levels of glycochenodeoxycholic acid (GCDCA) and GUDCA, but lower levels of isolithocholic acid compared to healthy controls. Interestingly, levels of the known neurotoxic bile acids glycocholic acid (GCA) and GCDCA were elevated in symptomatic HD patients, while levels of neuroprotective bile acids such as UDCA and TUDCA were higher in presymptomatic HD carriers, indicating a compensatory response to early neuronal stress and the potential involvement of different species of bile acids in disease mechanisms [73]. Moreover, bile acids may act as mediators in the gut–brain axis, with the gut microbiome influencing bile acid composition and signaling, potentially affecting HD progression. Although clinical trials for TUDCA in HD are limited, a Phase I study of ursodiol (UDCA), the precursor of TUDCA, showed it was safe and measurable in serum and cerebrospinal fluid of HD patients, laying groundwork for future therapeutic exploration (ClinicalTrials.gov; ID NCT00514774).

These findings reveal bile acids GCDCA, GUDCA, and isolithocholic acid could potentially serve as markers to distinguish between HD stages and healthy individuals, and support the hypothesis that bile acid dysregulation contributes to HD pathology and that bile acid-based therapies, particularly TUDCA, hold promise as a neuroprotective agent.

## 8. Therapeutic and Translational Potential

**Figure 2:**
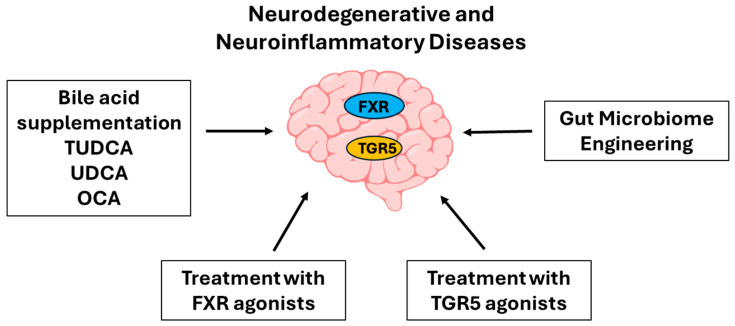
Potential therapeutic strategies targeting bile acid signaling in neurodegenerative and neuroinflammatory diseases. Approaches include bile acid supplementation (e.g., TUDCA, UDCA, OCA), gut microbiome engineering, and pharmacological activation of bile acid receptors FXR and TGR5 using specific agonists.

The growing recognition of bile acids as key modulators of neuroinflammation and CNS function has unlocked a wealth of therapeutic (Figure 2 and Table 2) and translational opportunities in neurology and immunology. Direct bile acid therapies, such as TUDCA, UDCA, and Obeticholic Acid (OCA), have demonstrated neuroprotective and anti-inflammatory anti-apoptotic effects in various neurodegenerative disease models, with TUDCA and UDCA showing promise in conditions like Alzheimer’s, Parkinson’s, and multiple sclerosis due to their ability to reduce microglial activation and oxidative stress [102,103,104,105,106]. Beyond supplementation, targeting bile acid receptors FXR and TGR5 offers a more precise strategy to modulate neuroimmune signaling, with FXR agonists suppressing inflammatory pathways and TGR5 agonists enhancing neuroprotection through cAMP-mediated mechanisms [107]. The gut microbiome’s influence on bile acid composition has also spurred interest in microbiome engineering, where probiotics and engineered bile salt hydrolases (BSHs) are being explored to cultivate a bile acid profile conducive to CNS health [108]. Furthermore, bile acid profiling in serum and CSF is emerging as a valuable tool in precision neurology, offering potential biomarkers for disease activity, progression, and therapeutic response in disorders such as multiple sclerosis, Alzheimer’s disease, and age-related neuroinflammation [79,87,109]. Together, these advances underscore the multifaceted potential of bile acid biology in shaping future interventions for inflammatory CNS disorders.

## 9. Knowledge Gaps and Future Directions

Despite the increasing recognition of bile acids as modulators of neuroinflammation and CNS pathology, several critical gaps continue to hinder both mechanistic insight and translational advancements, stressing the need for targeted research to bridge these divides and propel bile acid science from exploratory molecules to one of clinical relevance. Emerging research indeed suggests bile acids act as neuroactive signaling molecules, influencing neuroinflammatory pathways through bile acid receptors [44,49,123]. Future preclinical directions may include synthesis and characterization of bile acid derivatives or mimetics that selectively target these receptors in the CNS. Such compounds could be evaluated for their potential to attenuate neuroinflammation, promote neurogenesis, or enhance neuronal resilience across a range of experimental platforms, including in vivo rodent models, in vitro cellular assays, and advanced organoid systems that mimic human brain architecture. In parallel, the gut microbiota’s role in bile acid metabolism is gaining significant traction, particularly in the context of neuroimmune regulation. Dysbiosis, an imbalance in microbial composition, can lead to altered bile acid profiles, which in turn may exacerbate neuroinflammatory conditions by disrupting the gut–brain axis [124,125]. This has prompted a surge in interest toward animal models that incorporate microbiota–gut–brain dynamics, enabling researchers to dissect the physiological relationships between microbial metabolites, bile acid signaling, and neuroinflammation, and to identify microbial taxa or metabolic pathways that could be targeted to restore microbiota–gut–brain homeostasis. Another promising avenue of preclinical research involves the application of organotypic brain slice cultures that replicate disease-relevant neuroinflammatory responses, offering a translational platform for testing bile acid-based interventions [126,127]. These cultures preserve the cytoarchitecture and cell-type diversity of the brain, allowing for precise interrogation of bile acid-based interventions under disease-relevant conditions. By integrating these diverse preclinical approaches, scientists are well-positioned to uncover novel therapeutic strategies that target bile acid signaling to combat neuroinflammatory diseases.

One other major challenge lies in the incomplete mapping of bile acid receptors within the human brain; their precise localization and expression across human CNS cell types, including neurons, astrocytes, microglia, and endothelial cells, remain poorly defined, although these receptors are well-characterized in peripheral tissues. Employing advanced transcriptomic, proteomic, and spatial imaging techniques could reveal receptor distribution patterns and their variation across brain regions and disease states, thereby widening our understanding of bile acid signaling in neurological contexts [128]. Another pressing need is the implementation of longitudinal metabolomic studies in neurodegenerative diseases to potentially uncover early bile acid biomarkers or therapeutic windows that are missed by cross-sectional studies [129]. Moreover, integrating serum, CSF, and fecal bile acid profiles over time could reveal whether bile acid dysregulation precedes symptom onset, aligns with disease progression, or reflects therapeutic response. Additionally, the mechanisms by which bile acids traverse the BBB remain largely unexplored despite their presence in CSF and brain tissue. Investigating the roles of transporters and bile acid-specific carriers in BBB permeability could uncover novel strategies for regulating bile acid entry into the CNS and modulating neuroimmune interactions [130]. Compounding these issues is the lack of standardized protocols for CNS bile acid measurement. In this context, establishing rigorous standards for sample collection, storage, extraction, and mass spectrometry calibration, along with defining reference ranges for bile acid species in healthy and diseased CNS tissue, would greatly enhance biomarker development and clinical translation. Addressing these multifaceted challenges through a multidisciplinary perspective encompassing neuroscience, immunology, metabolomics, and microbiome science will be essential for unlocking the full therapeutic and diagnostic potential of bile acids in CNS disorders.

## 10. Conclusions

Bile acids have transcended their classical role as digestive surfactants to emerge as dynamic neuroimmune integrators. Through interactions with canonical and non-canonical receptors, their influence on glial and neuronal function, and their capacity to traverse the BBB, bile acids are now recognized as key modulators of neuroinflammation and CNS homeostasis. This review highlighted the currently known mechanistic links between bile acid signaling and neuroimmune pathways, the relevance of bile acid dysregulation across several neurological disorders, and the growing evidence for their therapeutic and diagnostic utility. Agents such as TUDCA, UDCA, and receptor-specific modulators show promise in mitigating neuroinflammatory damage, while bile acid profiling in serum and CSF offers a novel biomarker strategy for disease monitoring and risk stratification. Continued research into bile acid metabolism, receptor biology, and CNS delivery mechanisms will pave the way for innovative treatments that bridge the gap between gut and brain. Such research holds profound significance, not only for deepening our understanding of gut–brain communication, but also for unlocking new therapeutic avenues in neurodegenerative and neuroinflammatory diseases where current interventions remain limited. By elucidating the multifaceted roles of bile acids, future studies may redefine clinical approaches to diagnosis, prognosis, and personalized treatment in neurology.

## Figures and Tables

**Table 1:** Bile Acid Dysregulation Across Neurological and Neuroimmune Disorders.

Disorder	Key Features	Altered Bile Acids	Receptors Involved	Pathogenic Mechanisms	References
Alzheimer’s Disease	Amyloid-beta deposition, tau pathology	↑ DCA, TDCA, GDCA; ↓ CDCA, TUDCA	FXR, TGR5	Neuroinflammation, oxidative stress, gut–brain signaling	[66,67,68]
Parkinson’s Disease	Cognitive decline, motor symptoms	↓ CDCA, CA, UDCA, TUDCA; ↑ DCA, LCA	FXR, TGR5	Mitochondrial dysfunction, inflammation, appendix bile acid shifts	[49,69]
Multiple Sclerosis	Demyelination, immune dysregulation	Altered serum/CSF bile acids	FXR, TGR5	Microglial modulation, AKT/NFκB signaling, immune cell trafficking	[61,70]
Hepatic Encephalopathy	Cognitive impairment, motor disturbances	↑ TCA	S1PR2, TGR5	Microglial activation, systemic bile acid toxicity	[51,53]
Aging and Cognitive Decline	Synaptic dysfunction, memory loss	↑ TβMCA	FXR	Microbial dysbiosis, neuroimmune signaling disruption	[71]
Amyotrophic lateral sclerosis	Motor neuron degeneration, muscle wasting	↑ LCA, CDCA; ↓ UDCA	TGR5, FXR	Neuroinflammation, mitochondrial dysfunction, bile acid neurotoxicity	[55,72]
Huntington’s Disease	Chorea, psychiatric symptoms, cognitive decline	↓ CDCA, CA, UDCA; ↑ DCA	FXR, TGR5	Impaired bile acid metabolism, neuroinflammation, gut–brain axis disruption	[73]

↑ indicates increases and ↓ indicates decreases.

**Table 2:** Therapeutic Potential of Bile Acid-Based Interventions.

Compound	Target Disorders	Mechanisms of Action	Preclinical Insights	Clinical Insights
TUDCA	Alzheimer’s, Parkinson’s, MS, ALS	Anti-inflammatory, anti-apoptotic, antioxidant; neuroprotective	Anti-apoptotic and mitochondrial protection, reduction in ER stress; improved neuronal survival; reduced Experimental Autoimmune Encephalomyelitis (EAE) scores [61]; decreased Aβ deposition in Alzheimer’s Disease models [24,110]; attenuated autophagy in Parkinson’s disease [105].	Safe in ALS Phase II trials; Phase III showed no significant benefit but good tolerability [111]; changes in circulating T cells and the gut microbiota in MS patients [70].
UDCA (Ursodiol)	Alzheimer’s, Parkinson’s, MS, ALS, HD	Anti-inflammatory	attenuation of the production of pro-inflammatory cytokines and nitric oxide via inactivation of NF-kappaB in Alzheimer’s disease [112]; Neuroprotective effects in vitro and in vivo [82,113]; modulates bile acid receptors and apoptosis [114]; Mitochondrial stabilization [115]; microglial modulation via TGR5 [91,116].	Safe and well-tolerated in Parkinson’s Phase II trial (UP Study); improved mitochondrial function and gait (ClinicalTrials.gov ID NCT03840005); Ursodiol in Huntington’s Disease (NCT00514774).
GUDCA	ALS	Anti-apoptotic and anti-inflammatory	reduces matrix metalloproteinase-9 and caspase-9 activation [60].	-
Obeticholic acid/ 6-ECDCA (FXR agonist)	Parkinson’s Disease, Hepatic Encephalo-pathy	Anti-inflammatory and neuroprotective	inhibited astrocyte activation and neuroinflammation in a CEBPβ/NF-κB dependent manner [117]; suppresses pro-inflammatory cytokines and reduces C cell populations [43].	-
GPBAR1/ TGR5 agonists (TUDCA, INT-777)	Hepatic Encephalo-pathy, Parkinson’s Disease	Anti-inflammatory and neuroprotective	Alleviates neuroinflammation by altering neuron and microglia paracrine signaling [51]; reduces neuroinflammation and microglial cell activation [46]; mitigates neuropathic pain by reducing neuroinflammation [52]; alleviates neuroinflammation via Pellino3 inhibition of caspase-8/NLRP3 [118], modulating Mitochondrial Dynamics in Microglia [119], reducing microglia activation [44]; modulates NF-κB signaling [56].	-
AMX0035 (TUDCA combined with phenylbutyrate)	ALS	Neuroprotective	TUDCA administration delayed muscle denervation and reduced ER stress [95].	Reduced disease progression and longer survivability [120] (clinical trials NCT04987671, NCT03127514, NCT03488524, NCT05286372 and NCT04516096), marketed as a new ALS drug [121].
Cholestyramine	Hepatic Encephalopathy and cognitive decline	Anti-inflammatory; Bile acid chelation; cytokine modulation	Reduced IL-1β/IL-6; increased IL-10 and TGR5 expression in animal models [27]; diminishes brain inflammatory signaling [122]; reduces pro-inflammatory cytokine expression in the cortex [53].	Used clinically for hepatic encephalopathy and NAFLD; combination trials with elobixibat show safety and efficacy.
Microbiota Modulation	Aging and Cognitive Decline	Anti-inflammatory; alters bile acid profiles; reduces TβMCA	Preserves cognition, reduces neuroinflammation, improves SCFA production [77].	Emerging clinical interest; microbiome-targeted therapies under development for mild cognitive impairment and dementia.

## Data Availability

No new data were created or analyzed in this study. Data sharing is not applicable to this article.

## Abbreviations

The following abbreviations are used in this manuscript:

ADT	Androgen deprivation therapy
A2M	Alpha-2 macroglobulin
AHSG	Alpha-2 HS glycoprotein
ALB	Albumin
ALS	Amyotrophic lateral sclerosis
APOA1	Apolipoprotein A1
APOH	Apolipoprotein H
AKT/NFκB	Ak strain transforming/nuclear factor kappa B (signaling pathway)
BBB	Blood–brain barrier
BSH	Bile salt hydrolases
CSF	Cerebrospinal fluid
CNS	Central nervous system
CDCA	Chenodeoxycholic acid
CA	Cholic acid
cAMP	Cyclic adenosine monophosphate
DCA	Deoxycholic acid
ERK1/2	Extracellular signal-regulated kinase 1/2
EAE	Experimental autoimmune encephalomyelitis
FXR	Farnesoid x receptor
GPBAR1	G-protein coupled bile acid receptor 1 (GPBAR1 aka TGR5)
GPCR	G-protein coupled receptor
GCA	Glycocholic acid
GDCA	Glycodeoxycholic acid
GUDCA	Glucoursodeoxycholic acid
GCDCA	Glycochenodeoxycholic acid
HD	Huntington’s disease
HE	Hepatic encephalopathy
IL-1β	Interleukin-1 beta
IL-6	Interleukin-6
IL-10	Interleukin-10
LCA	Lithocholic acid
MS	Multiple sclerosis
MG6	Microglial cells
OATP	Organic anion transport polypeptide
OCA	Obeticholic acid
PBC	Primary biliary cirrhosis
ROS	Reactive oxygen species
SCFA	Short-chain fatty acid
SPP2	Secreted phosphoprotein 2
S1PR2	Sphingosine-1-phosphate receptor 2
TCA	Taurocholic acid
TDCA	Taurodeoxycholic acid
TUDCA	Tauroursodeoxycholic acid
TβMCA	Tauro-beta-muricholic acid
TGR5	Takeda G-protein coupled receptor 5
TNFα	Tumor necrosis factor alpha
UDCA	Ursodeoxycholic acid
6-ECDCA	6alpha-ethyl-chenodeoxycholic acid
